# Drug response profiling can predict response to ponatinib in a patient with t(1;9)(q24;q34)-associated B-cell acute lymphoblastic leukemia

**DOI:** 10.1038/bcj.2015.13

**Published:** 2015-03-13

**Authors:** Y Collette, T Prébet, A Goubard, J Adélaïde, R Castellano, N Carbuccia, S Garnier, A Guille, C Arnoulet, A Charbonier, M J Mozziconacci, D Birnbaum, M Chaffanet, N Vey

**Affiliations:** 1 Centre de Recherche en Cancérologie de Marseille (CRCM) Inserm UMR 1068, Institut Paoli-Calmettes, Aix-Marseille Université UM 105, CNRS UMR 7258, Marseille, France; 2 Département d'hématologie, Institut Paoli-Calmettes, Aix-Marseille Université UM 105, CNRS UMR 7258, Marseille, France

Tyrosine kinase inhibitor (TKI)-based targeted therapy has significantly modified the outcome for patients with chronic myeloid leukemia (CML) in chronic phase. However, resistance remains a major concern in blastic phase of CML and in Philadelphia chromosome positive B-cell acute lymphoblastic leukemia (Ph+ B-ALL). Second- and third-generation TKIs have been developed to overcome resistance to first generation drugs, but selecting the appropriate drug has become a challenge. Various tests are available to determine a patient's disease status in CML including the mechanisms of resistance when involved, but clinical experience is limited in ALL, especially those with poorly defined *ABL1* rearrangements. Here, we report a case of ALL associated with a t(1;9)(q24;q34) *RCSD1*-*ABL1* rearrangement. We show how *ex vivo* drug response profiling (DRP) may help choose among various therapeutic options.

A 26 year-old female presented in September 2012 with fatigue and antibiotic-resistant ENT infection. Blood counts showed WBC 26 g/l with 84% blasts on peripheral blood. Marrow smear examination confirmed the diagnosis of ALL with 86% blasts. Flow cytometry was consistent with common ALL with expression of CD79a^+^, CD19^+^, CD10^+^ and weak expression of CD20. Positivity of CD33 was also noticed. Cytogenetics showed an uncommon t(1;9)(q24;q34) and involvement of *ABL1* was confirmed by fluorescence *in situ* hybridization FISH (not shown). In agreement with the previous characterization of t(1;9)(q24;q34),^1, 2, 3^ Real time-PCR analysis from leukemic cells showed the expression of the *RCSD1-ABL1* gene fusion (Supplementary Material 1a), and sequencing of the PCR products showed that *RCSD1* exon 3 was fused to *ABL1* exon 4 (Supplementary Material 1b).

On the basis of these results and the cases previously reported^2^ the patient was treated with steroids, vincristine and dasatinib. In October 2012, the patient reached morphologic complete remission (CR) and FISH analysis showed a 2% residual disease on 500 nuclei. She received consolidation based on hyperCVAD dasatinib strategy and 0.2% residual disease by FISH was achieved after two cycles of consolidation. A matched-sibling hematopoietic stem cell (HSC) transplantation with reduced toxicity conditioning was performed in February 2013. The patient relapsed (R1) in December 2013. Clonal evolution was observed with newly appearing t(6;13) (35% of mitosis) and t(11;15) (8% of mitosis) in addition to the t(1;9). She was treated with intensive chemotherapy (mitoxantrone, high dose cytarabine, etoposide, dexamethasone and asparaginase). She achieved a second CR in January 2014, received consolidation chemotherapy but relapsed (R2) in April 2014 with 10% marrow blasts.

An *ex vivo* functional evaluation of the DRP of the patient leukemic blasts at R1 was undertaken using our DRP platform^4^ with a specific focus on TKIs. Each drug was tested over a 1000-fold concentration range, allowing the establishment of dose-response curves and the identification of half-maximal effective concentration responses (Figures 1a, b and Supplementary Material 2). Resistance and/or low response to some TKIs were observed such as dasatinib or nilotinib (EC50> or ≅20 μM), whereas measurable responses to others were noted, notably to ponatinib (EC50=0.8 μM). This differential response to ponatinib as compared with dasatinib was confirmed in a secondary dose-response study (Figure 1c), which showed comparable drug sensitive profile for samples of the patient obtained at diagnosis and at R1. Exon sequencing did not show any *ABL1* mutation that could account for the differential response to the two drugs. A total of 419 other genes were studied (Supplementary material 3); none was differentially mutated.

In parallel, the samples of the patient collected at diagnosis and at R1 were grafted into NSG mice. Engraftment was successful for both samples, starting at day 57 post-injection for samples obtained at R1 (74.7±16.1 blasts/μl of blood) and at day 72 post-injection for samples obtained at diagnosis (11.4±3 blasts/μl of blood, as compared with 146±58.6 blasts for samples obtained at R1), and both progressed then after (Figure 1d). Genomic profiles of leukemic cells from both the patient and the cognate transplants were established by using array-comparative genomic hybridizations. They were similar (Supplementary Materials 4a vs 4c and 4b vs 4d, respectively) indicating that the generated animal models reflected the disease. Interestingly, 6p23-p24 and 13q14.2-q14.3 regional losses occurred in leukemic cells from both the patients at R1 and the corresponding cognate transplants (Supplementary Materials 4b and 4d, respectively) suggesting a genomic evolution of the disease. Differences of gene copy number variations in these chromosomal regions are illustrated for the four samples (Supplementary Materials 4e and 4f). They targeted several genes including potential candidates such as *JARID2* (on 6p22.3), *miRNA15* and *miRNA16* (on 13q14.2, arrows). These losses are likely to be associated with the t(6;13)(p23;q14) event characterized in leukemic cells from the patient at R1 (Supplementary Material 4g).

One mouse from the group of animals transplanted with the relapse sample was killed at day 106 and bone marrow cells were transferred to secondary recipients (10^6^ cells per mice). Fifty days after engraftment, blasts were counted and animals were homogeneously distributed into three groups receiving diluent (dimethyl sulfoxide), dasatinib (25 mg/Kg, 5 days out of 7 for 4 weeks) or ponatinib (30 mg/Kg, 4 days out of 7 for 4 weeks) as described.^5^ As shown in Figure 1e, at days 14 and 28 after initiation of treatment, blast counts were determined, showing reduced progression of the disease in dasatinib-treated animals (96±41 blasts/μl of blood) as compared with control animals (344±63 blasts/μl of blood) at day 28 (*P*<0.0001), and a significant regression in ponatinib-treated animals, both at day 14 (49±17 blasts/μl of blood vs 296±169 blasts/μl of blood in the control group, *P*<0.0001) and day 28 (19±7 blasts/μl of blood vs 344±63 blasts/μl of blood, *P*<0.0001). On the basis of this, the patient was started on treatment with ponatinib monotherapy (45 mg/day) in May 2014 and achieved a CR2. She received a second HSC transplant from a HLA-haplo-identical donor in July 2014 but relapsed at day 77 post transplantation with 12% BM blasts. Ponatinib was restarted at this time. BM smear at day 92 showed 4% blasts. Signs of severe graft versus host disease were present at this time. The patient died of a septic shock 2 weeks later.

This study illustrates how DRP may help select an appropriate disease-adapted TKI, including consideration of treatment changes, especially in case of rare and poorly defined leukemia such as RCSD1-ABL1 B-cell ALL. It illustrates the potential of DRP to predict treatment effects. *Ex vivo* testing was indeed confirmed by *in vivo* experiments using the patient's leukemic cells transplanted into NSG mice. In addition, clinical activity observed with ponatinib monotherapy that was offered to the patient after her second relapse on the basis of DRP ultimately confirmed the clinical relevance of the test.

To our knowledge, this is the first report of ponatinib successful treatment of a t(1;9)(q24 ;q34) B-cell ALL. The superior efficacy of ponatinib as compared with dasatinib on the patient's leukemia cells, both *ex vivo* and in our xenotransplantation model, was unexpected, based on the previous knowledge. Ponatinib is a third-generation TKI inhibitor designed to target the most frequent imatinib resistance-associated mutations in CML (such as the T315I substitution).^6^ *In vitro*, as compared with dasatinib, ponatinib exhibits significant superior inhibitory activity against mutated forms of BCR-ABL1, but is rather similar in efficacy against wild-type BCR-ABL1.^7, 8^ The differential response of the patient's leukemia cells to dasatinib as compared with ponatinib in our case was not explained by the presence of mutation(s) in *ABL1* coding sequences including T315I mutation, and is thus likely the result of either the distinct signaling capability of RCSD1-ABL1,^3^ the broad spectrum of ponatinib targets and/or the intrinsic spatial adaptability of the inhibitor to bind different conformational states of the kinase.^7, 8^ Indeed, the RCSD1-ABL1 fusion contains only a truncated ABL1 protein starting from the exon 4-encoded region, and thus results in a fusion protein that lacks the SH3 regulatory module and retains only part of the SH2 domain, which is involved in the regulation of the kinase activity of BCR-ABL1.^9^ This difference in structure may differentially impact dasatinib and ponatinib efficacy in contacting the active site of the ABL1 kinase. Current molecular studies are undertaken to study these hypothesis. Interestingly, ponatinib anti-leukemic activity was not affected by the occurrence of t(6;13) and t(11;15) at relapse.

More generally, this report, together with others^10, 11^ suggest that precision medicine may be better based on both genomic data and drug response (including *ex vivo*) data than on only one of these.

## Figures and Tables

**Figure 1:**
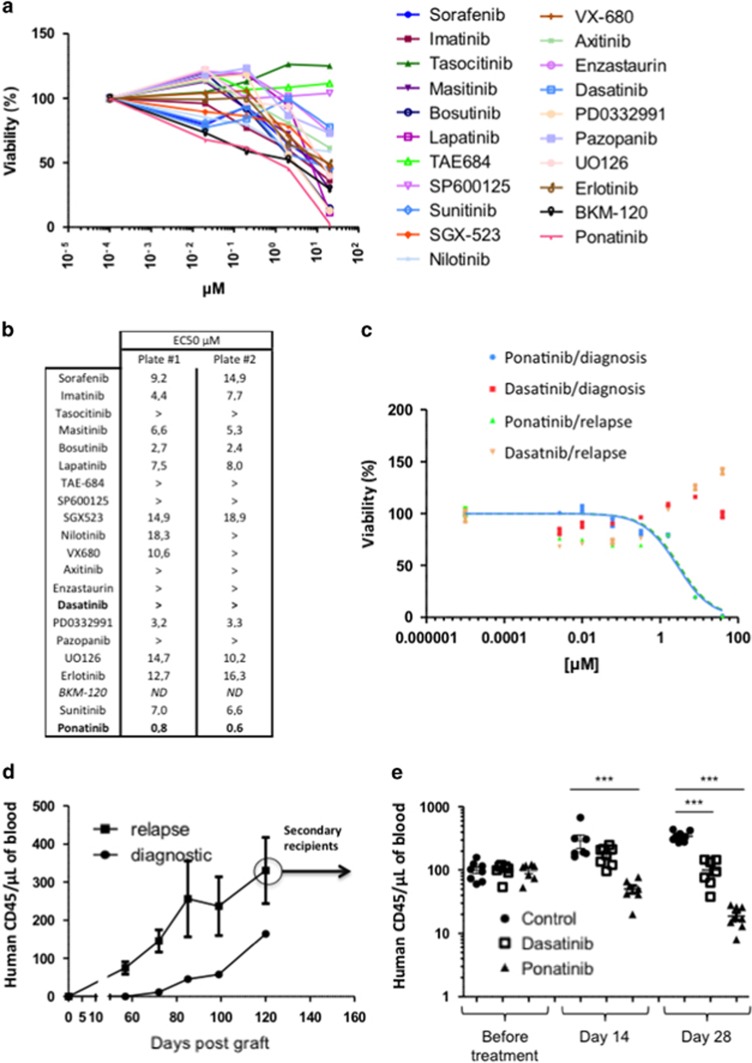
*Ex vivo* DRP Identification and *in vivo* validation of a t(1;9)(q24;q34) *RCSD1*-*ABL1* bearing patient sensitivity to ponatinib. (**a**) Ficoll-purified blood cells obtained from the patient at relapse (April 2014) were exposed for 48 h to varying concentrations of the indicated TKIs, and cell viability was assessed using CellTiter-Glo luminescent assay (Promega, Charbonnières les Bains, France). The data are presented as percent viability after normalization to negative control (dimethyl sulfoxide only). (**b**) Effective half-maximal concentration values (EC50) deduced from the dose-response curves obtained in duplicate from two distinct drug testing plates (plate #1 and plate #2) are shown. (**c**) As in **a**, except that Ficoll-purified blood cells obtained from the patient at diagnosis (September 2012) and at relapse (April 2014) were exposed for 48 h to varying concentrations of dasatinib and ponatinib. (**d**) Ficoll-purified blood cells obtained from the patient at diagnosis (September 2012) and at relapse (April 2014) were inoculated in the tail vein of NSG mice and the presence of human B-ALL blast cells in peripheral blood obtained by retro-orbital bleeding was determined by flow cytometry at different time points. Results are presented as the number of human CD45 positive cells per μl of mice blood (mean±s.e.m., 4 animals per group). (**e**) Secondary transplantation was conducted by injecting bone marrow cells of primary transplanted mice from **d** in new NSG recepients. At day 50 after secondary transplantation, human blasts present in mice blood were determined to homogeneously distribute recepient mice into three groups receiving vehicle, dasatinib (25 mg/kg, *po*) or ponatinib (30 mg/kg). Results are presented as the number of human CD45 positive cells per μl of mice blood as determined by Flow cytometry at day 14 and day 28 after initiation of treatment (mean±s.e.m., 8 animals per group). The data were analyzed with a one-way analysis of variance test (GraphPad Prism, GraphPad, La Jolla, CA, USA) and each group was further compared with the vehicle control group for statistical significance using Dunnett multiple comparison test. ***P<0.0001.
