# Supplementary figures and images for: Drug response profiling can predict response to ponatinib in a patient with t(1;9)(q24;q34)-associated B-cell acute lymphoblastic leukemia
Source: Blood Cancer J. 2015 Mar 13;5(3):e292–. doi: 10.1038/bcj.2015.13 (PMC4382656; doi:10.1038/bcj.2015.13)

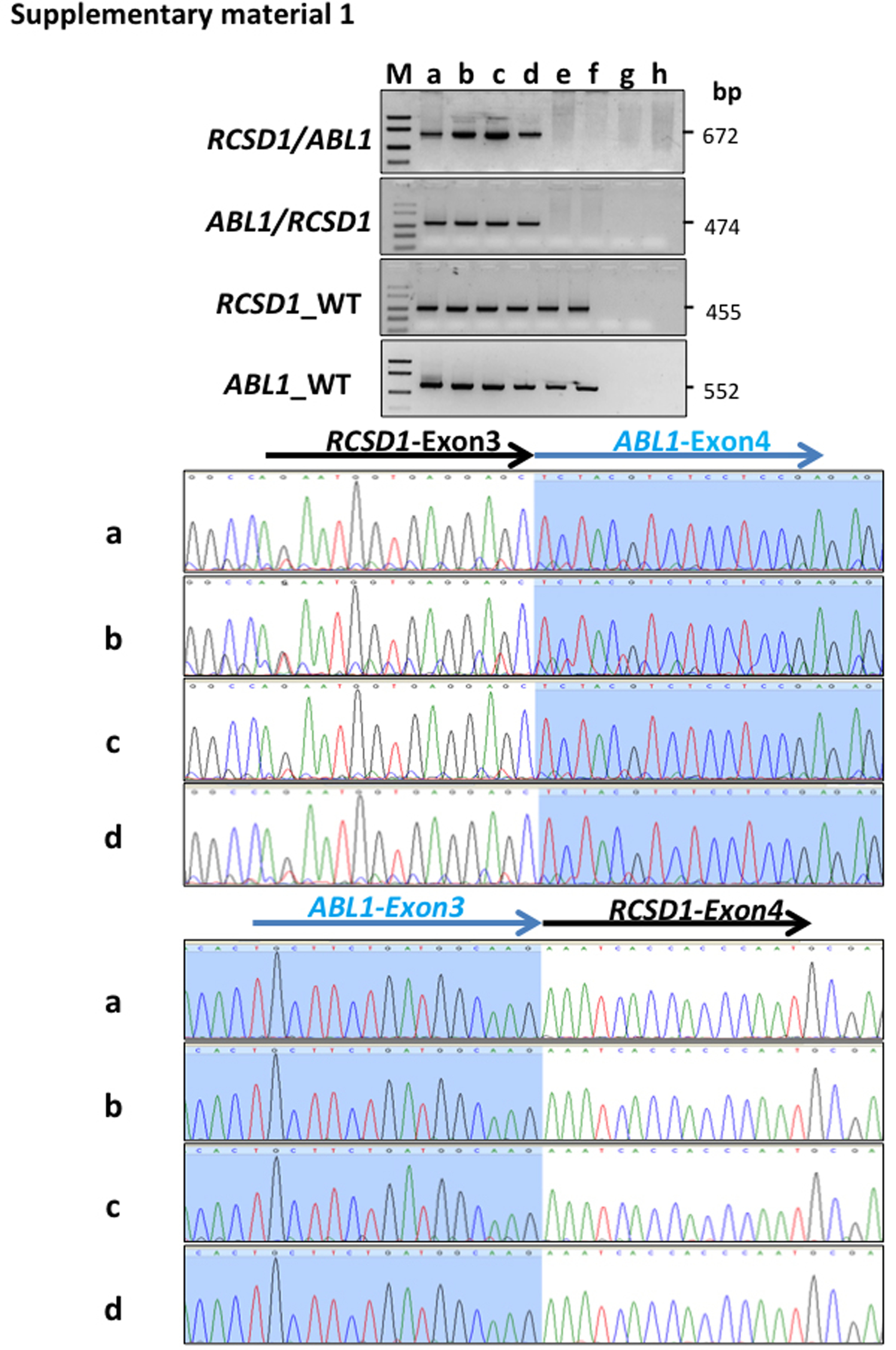

Supplement: Supplementary Figure 1 [file bcj201513x1.tif]

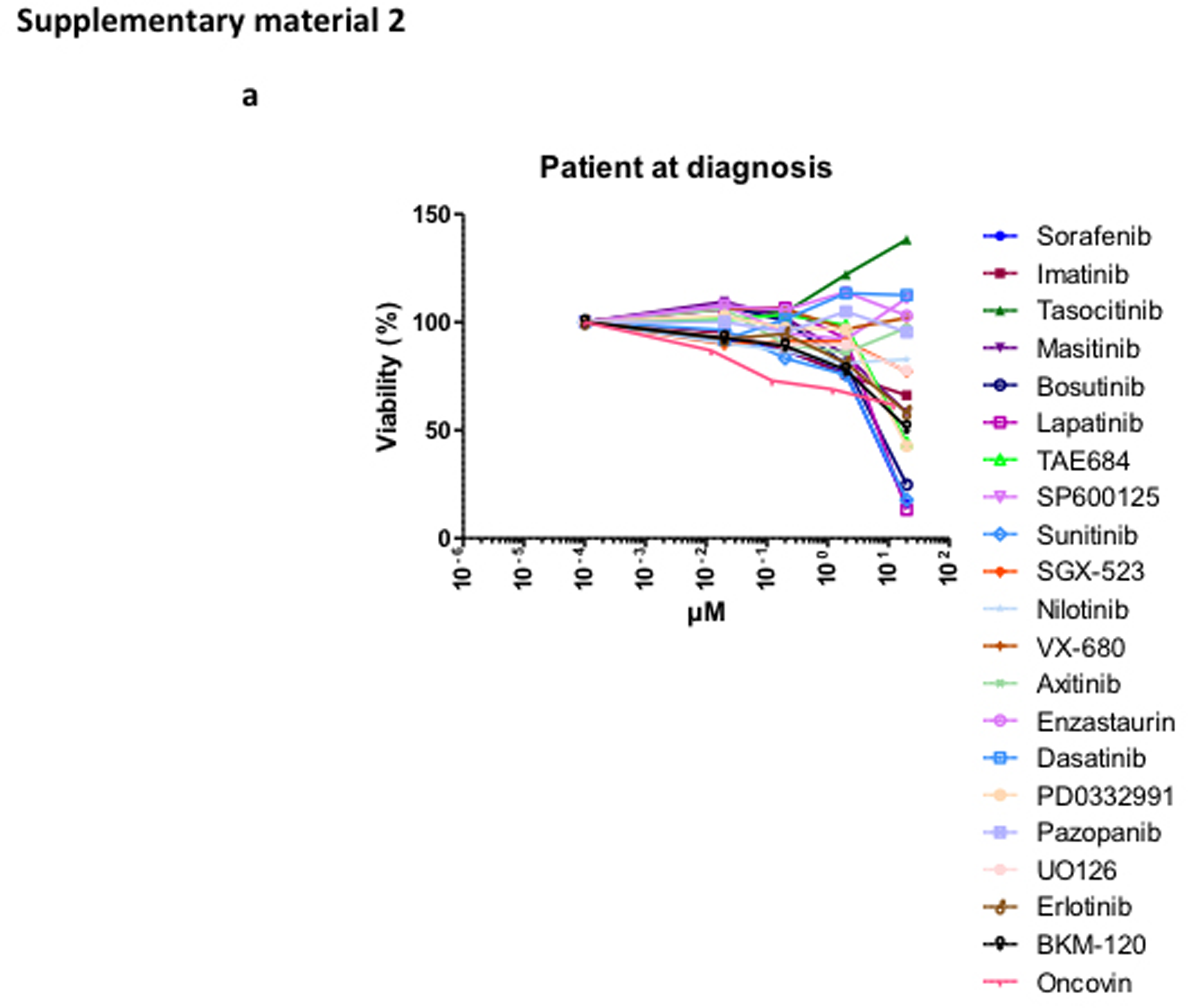

Supplement: Supplementary Figure 2 [file bcj201513x2.tif]

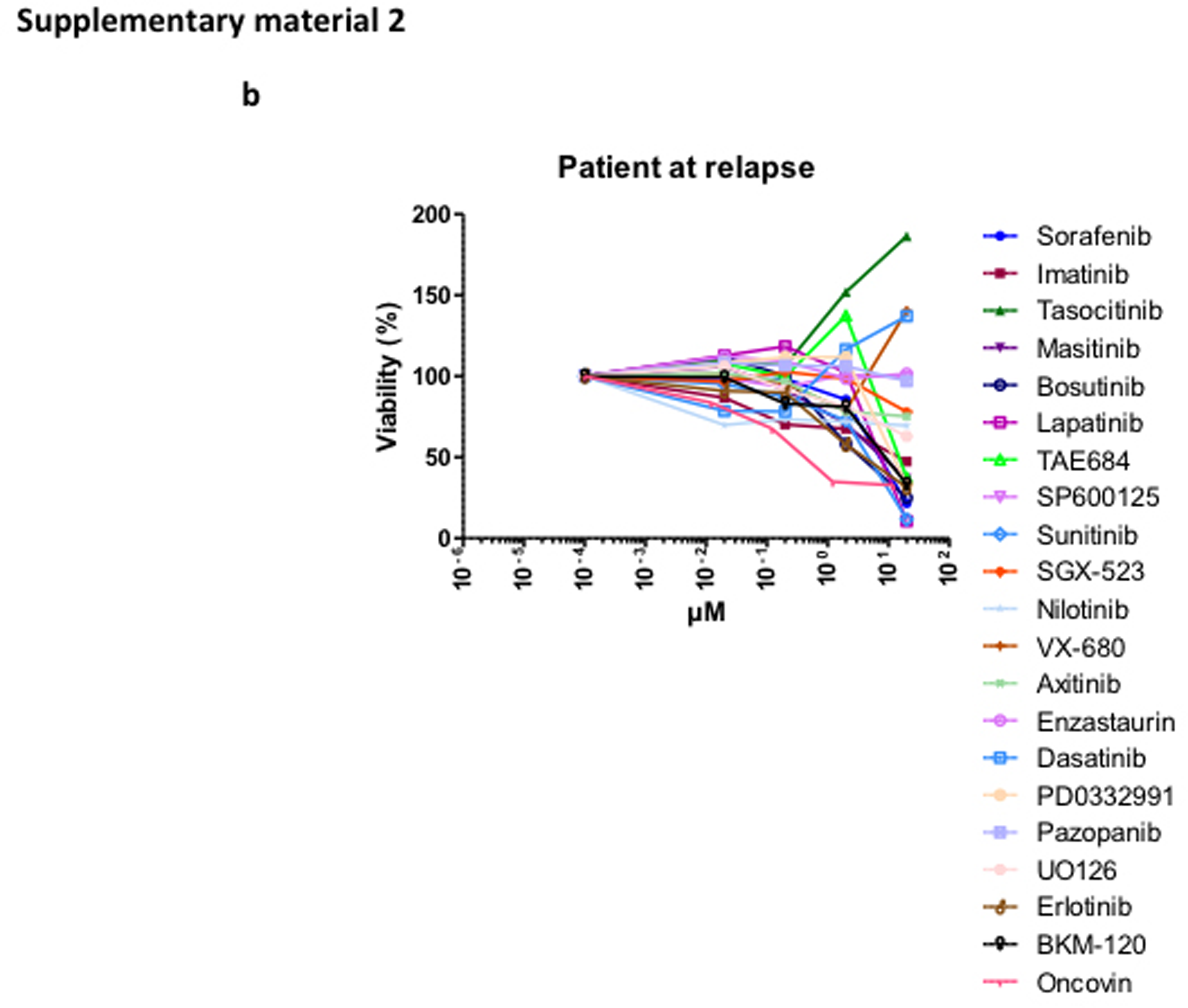

Supplement: Supplementary Figure 3 [file bcj201513x3.tif]

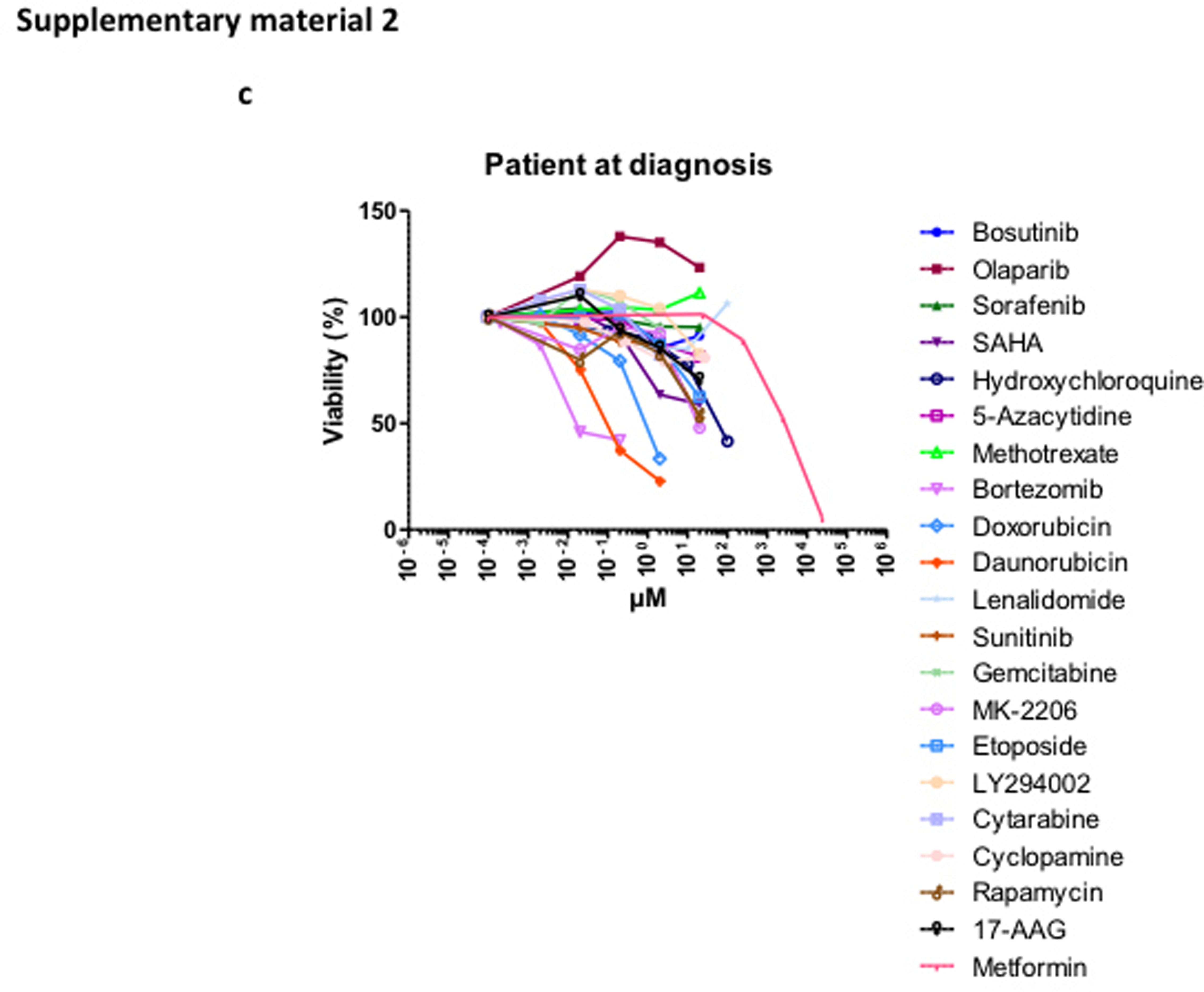

Supplement: Supplementary Figure 4 [file bcj201513x4.tif]

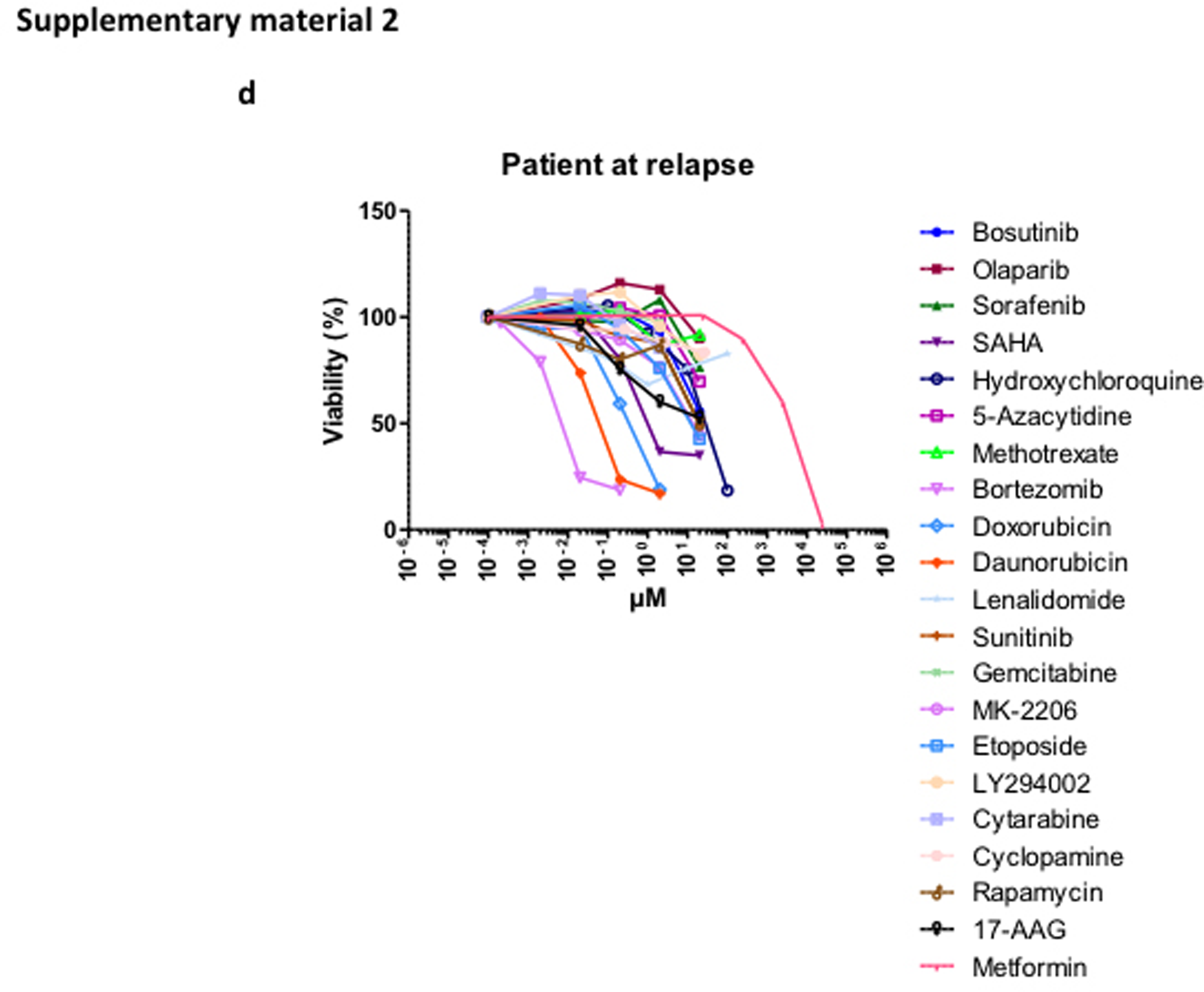

Supplement: Supplementary Figure 5 [file bcj201513x5.tif]

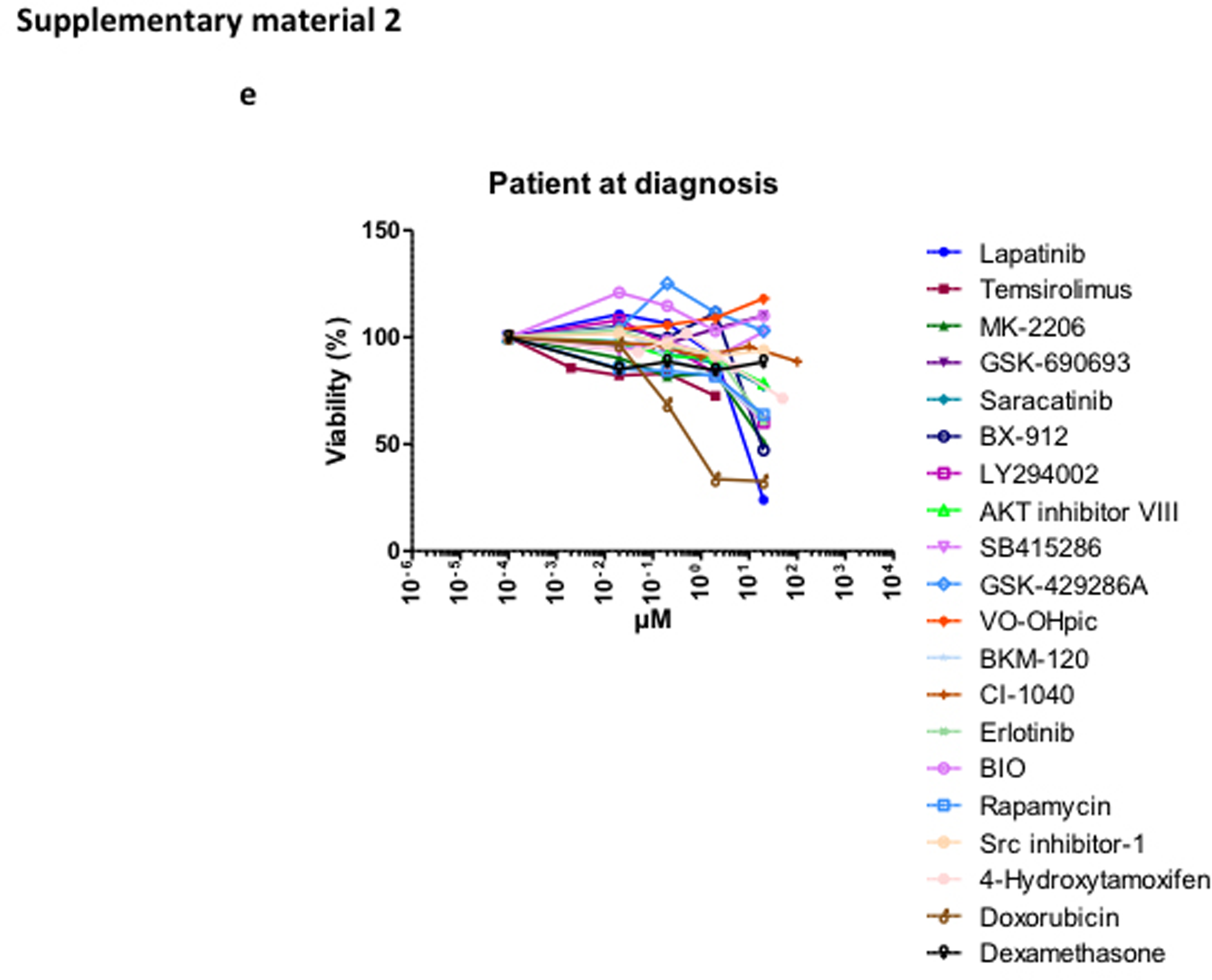

Supplement: Supplementary Figure 6 [file bcj201513x6.tif]

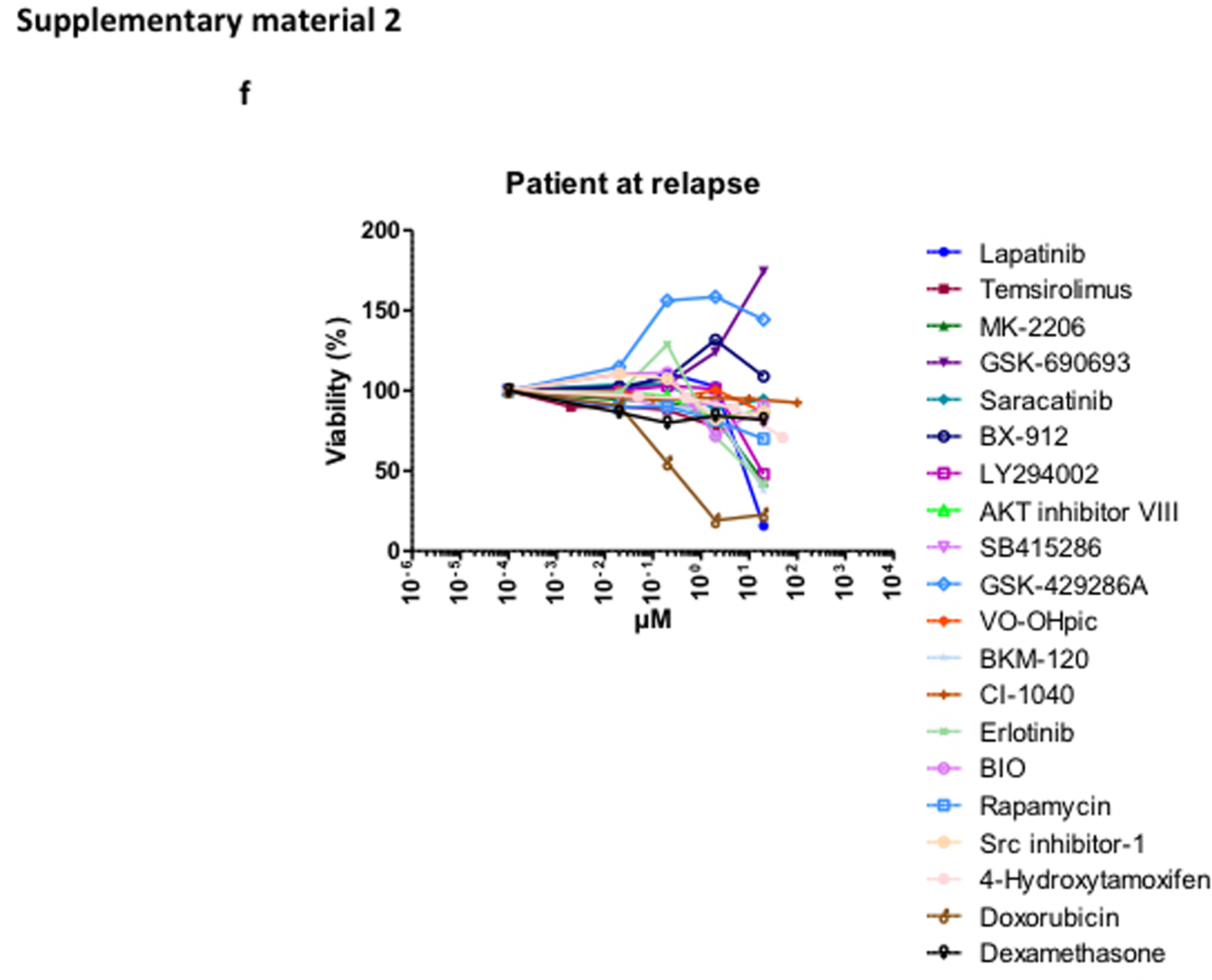

Supplement: Supplementary Figure 7 [file bcj201513x7.tif]

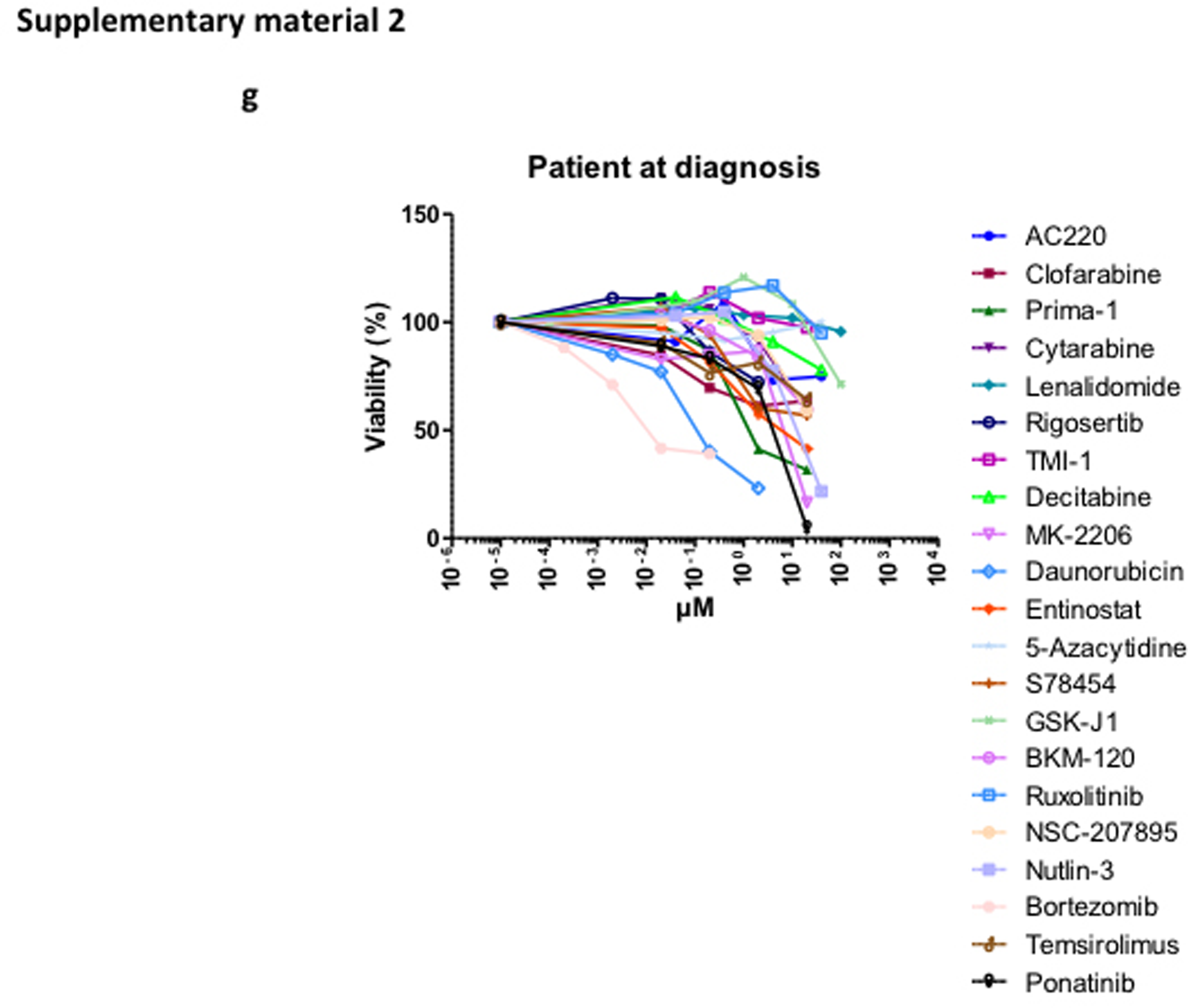

Supplement: Supplementary Figure 8 [file bcj201513x8.tif]

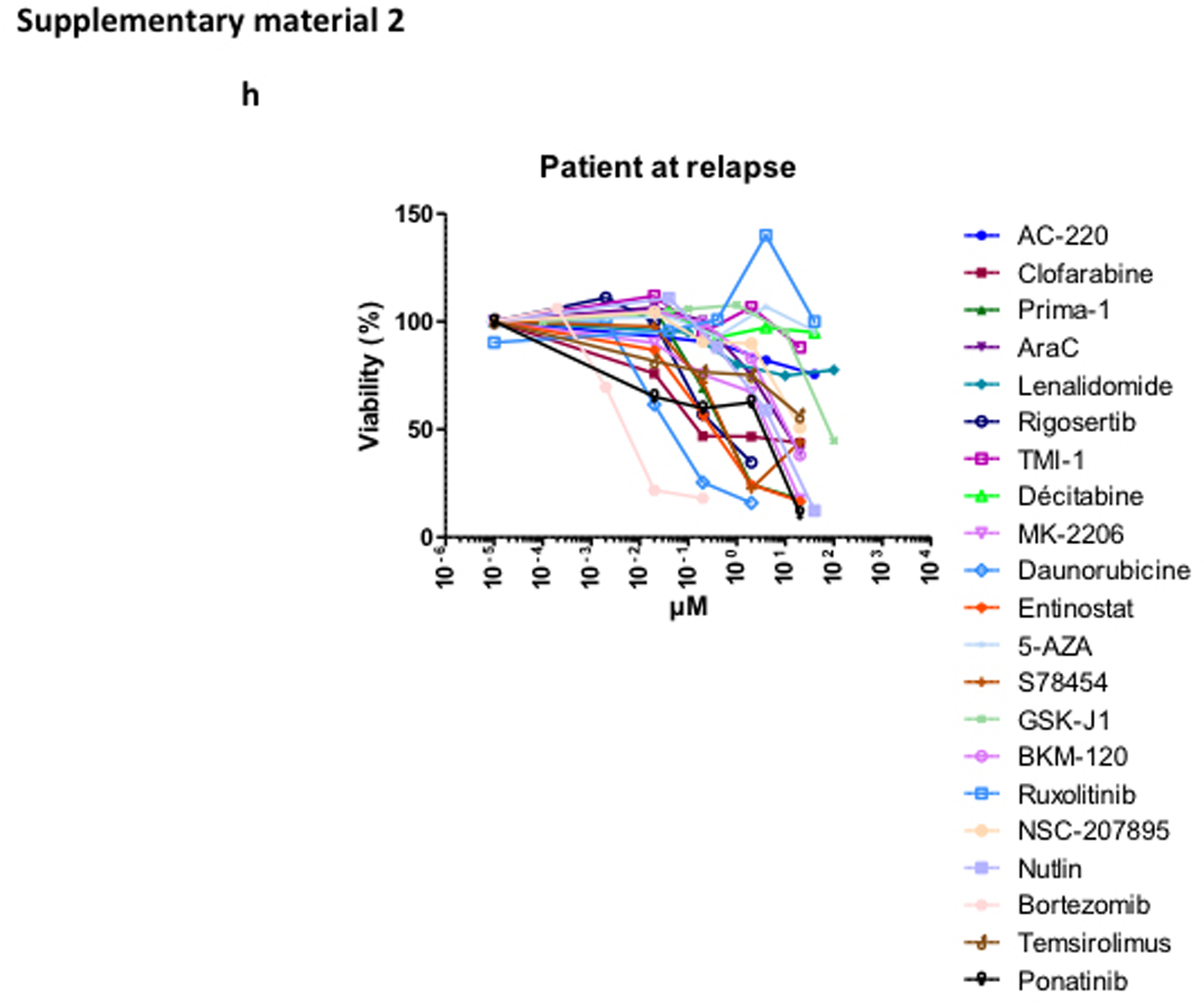

Supplement: Supplementary Figure 9 [file bcj201513x9.tif]

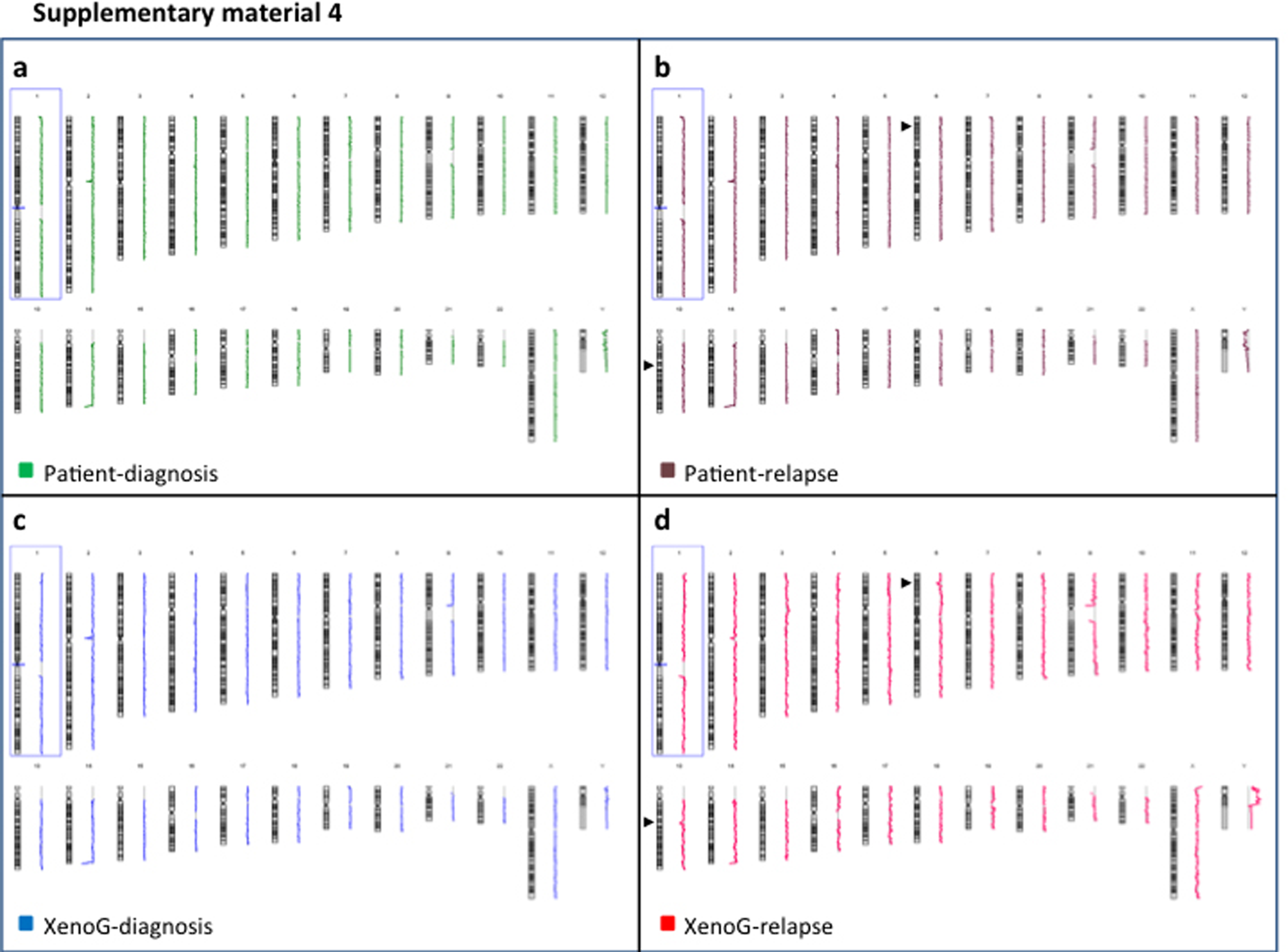

Supplement: Supplementary Figure 10 [file bcj201513x10.tif]

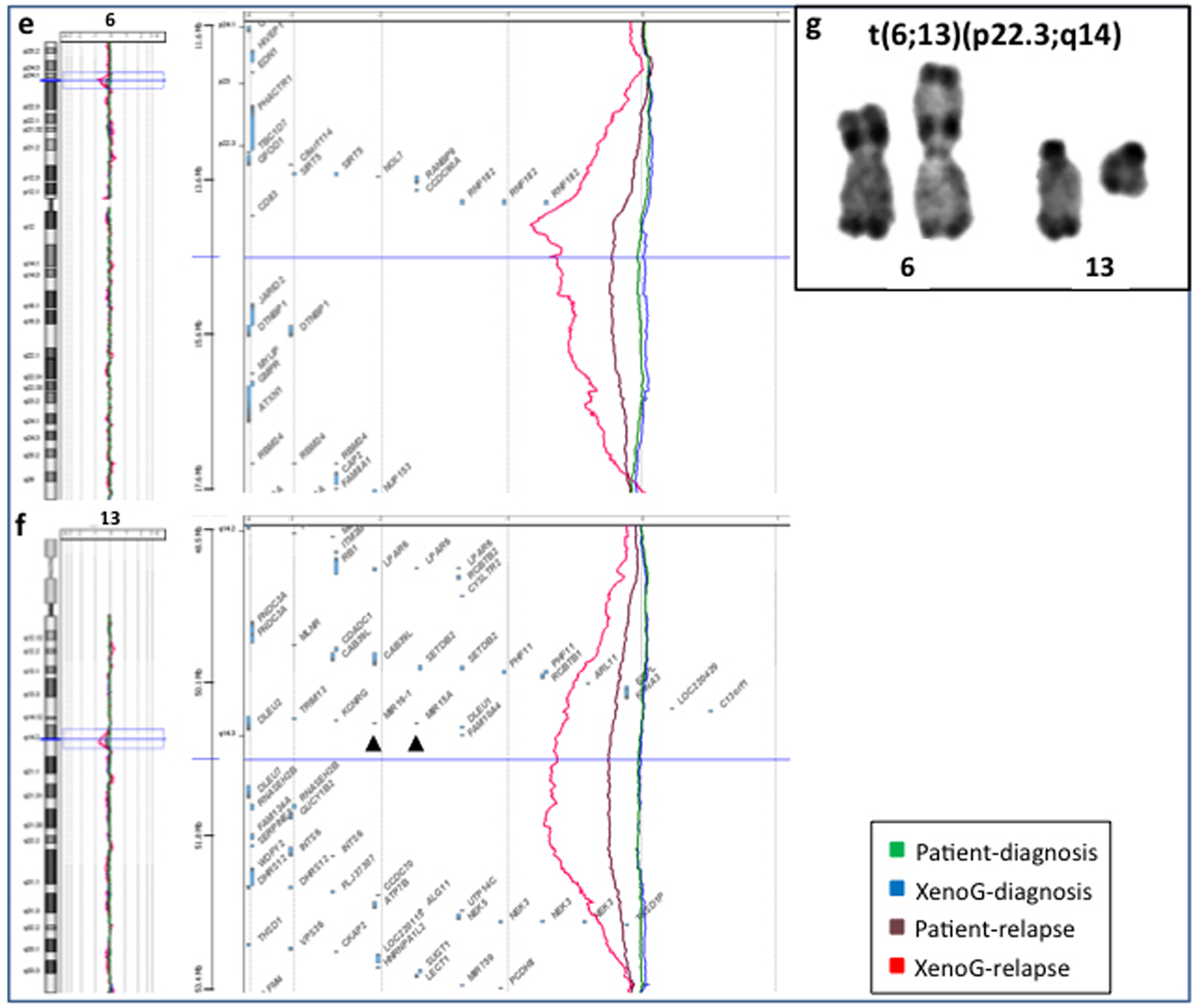

Supplement: Supplementary Figure 11 [file bcj201513x11.tif]

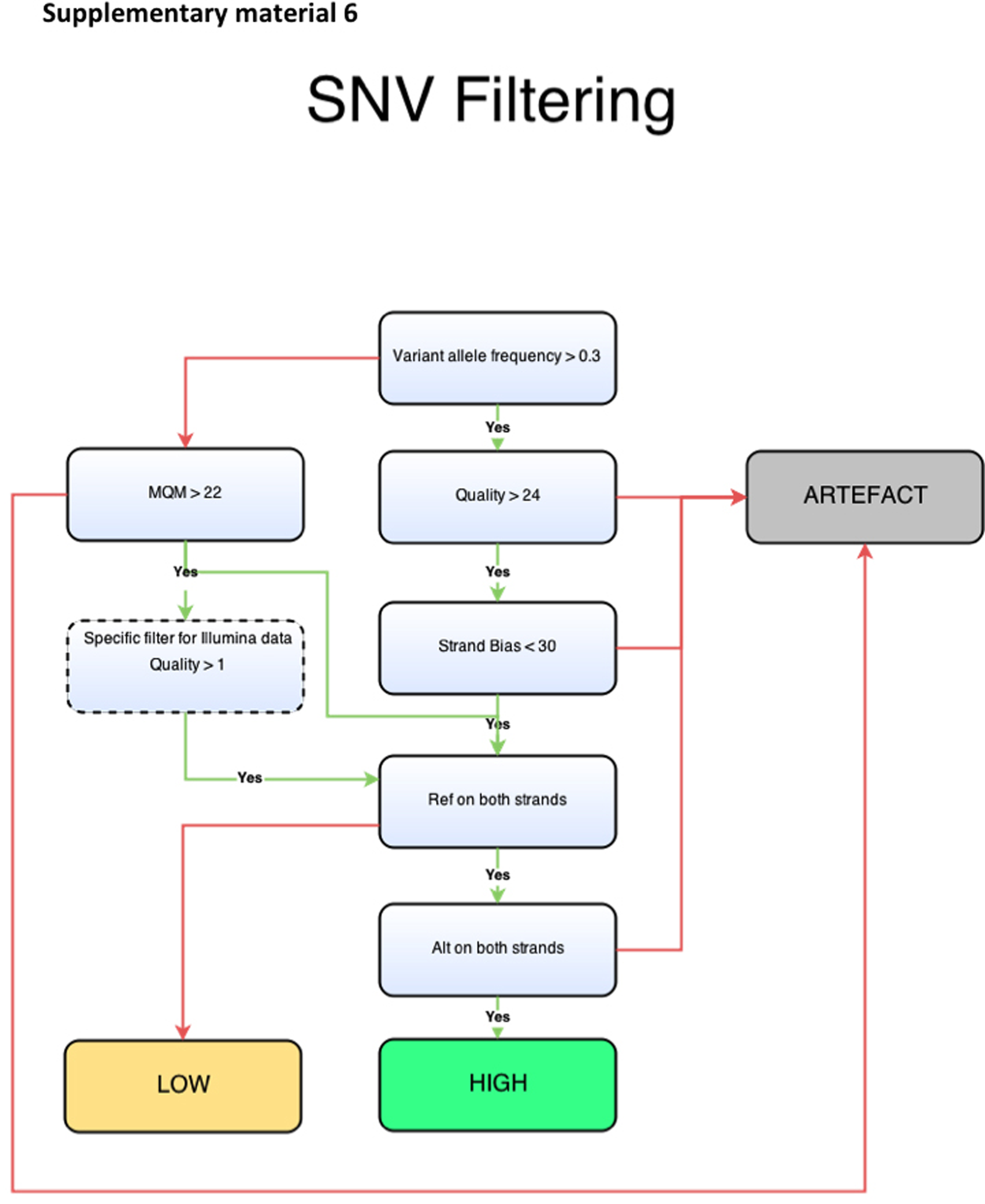

Supplement: Supplementary Figure 12 [file bcj201513x12.tif]
