# Supplementary material for: Drug response profiling can predict response to ponatinib in a patient with t(1;9)(q24;q34)-associated B-cell acute lymphoblastic leukemia
Source: Blood Cancer J. 2015 Mar 13;5(3):e292–. doi: 10.1038/bcj.2015.13 (PMC4382656; doi:10.1038/bcj.2015.13)
Supplement: Supplementary Information 1 [file bcj201513x15.docx]

**Supplementary material 7: Material and methods**

***Patient and ethics statement***

The patient has been treated at Institut Paoli Calmettes, Marseille, France. She gave her informed consent for the investigations described above and the use of clinical and biological data for scientific purpose. Use of DRP has been validated by the Internal Review Board of institut Paoli Calmette.

***Molecular analysis***

**PCR detection of *RCSD1-ABL1* gene fusion**

The *RCSD1-ABL1* gene fusion was detected by using PCR amplification of retrotranscribed mRNA (RT-PCR) from leukemic cells of the patients. Wild-type and fusion transcripts were amplified using combinations of *RCSD1* and *ABL1* primer sequences previously designed (Mustjoki *et al.*, 2009) and described in supplementary material 5. PCR products were visualized on agarose gel with ethidium bromide, and sequenced.

**Gene profiling**

DNAs were extracted as previously described (Adélaïde et al., 2007) and controled on Agilent Bioanalyzer (Agilent Technologies, Massy, France). Genomic profiles of the leukemic cells from the patient at diagnosis and at relapse as well as from their corresponding cognate transplants were established by using array-comparative genomic hybridization (aCGH) onto high-resolution 4x180K CGH microarrays (SurePrint G3 Human CGH Microarray Kit, Agilent Technologies, Massy, France). A Human genomic DNA female was used as reference (G152A, Promega). Both approaches and analysis methods have been used in our previous studies (Adélaïde et al., 2007; Sircoulomb et al., 2010; Bekhouche et al., 2011; Sircoulomb et al., 2011; Cornen et al., 2014). All probes for aCGH were mapped according to the hg19/NCBI human genome mapping database.

**HaloPlex target enrichment system**

For each sample, library of all coding exons and intron-exon boundaries of a total of 419 genes known to be associated with myeloid pathologies and solid tumors was prepared using a HaloPlex target enrichment kit (Agilent technologies, Santa Clara, USA), following the manufacturer's instruction. Briefly, 225 ng DNA was fragmented using restriction enzymes. The probe library was added and hybridized to the targeted fragments. Each HaloPlex probe is an [oligonucleotide](javascript:void(0);) designed to hybridize to both ends of a targeted DNA restriction fragment, thereby guiding the targeted fragments to form circular DNA molecules. The probe also contains a method-specific sequencing motif and that is incorporated during the circularization. Here, we used two HaloPlex libraries “hemato V7b” (Design ID: 22130-1386599097) and “CCP V6” (Design ID: 27066-1392300924) containing Ion Torrent PGM- and Illumina-specific sequencing motifs to respectively favorise target enrichment of 84 and 365 genes known to be associated with myeloid pathologies and solid tumors (Supplementary material 3). The HaloPlex probes were biotinylated and the targeted fragments were then retrieved with magnetic streptavidin beads. Next, the circular molecules are closed by ligation. At last, the enriched DNA fragments were amplified by PCR followed by deep sequencing on Ion Torrent PGM (Life Technologies) and Illumina MiSeq platforms.

**NGS analysis**

DNA sequencing was performed on a Ion Torrent PGM sequencing system (Life Technologies, Saint Aubin, France) with 250-bp, single-end reads and on a MiSeq (Illumina) with 2x150-bp, paired-end reads according to the manufacturer’s instructions. The read sequencing was aligned to human reference genome (UCSC hg19) (<http://hgdownload.cse.ucsc.edu/>) using Burrows-Wheeler Aligner ([Li and Durbin, 2009](http://www.sciencedirect.com/science/article/pii/S0197458014004722#bib12)). Samples were sequenced at about 600 and 300 x coverage with the PGM and illumina platforms, respectively. Bam files were processed according to the workflow recommended for variant analysis with GATK. Briefly, reads groups were added with PICARD tools version 1.91(1451) (http://broadinstitute.github.io/picard/). Local realignment and score recalibration were done using GATK version 2.5-2-gf57256b. SNVs calling was done with FreeBayes version 0.9.9 (Garrison and Marth, 2012) with a minimal alternate variant frequency and coverage set at 0.01 and 5 for PGM platform and 0.02 and 10 for Illumina Miseq platform.

Indel calling was done using GATK haplotype caller version 2.5-2-gf57256b (DePristo et al., 2011) with default parameters. The variants, i.e SNVs and Indel, were annotated with RefSeq annotation, dbsnp129, dbsn138NonFlagged, 1000 Genome and ESP6500 population frequencies, COSMIC V68, Clinvar, and predicted effects score on the protein using the Annotate Variation Software (ANNOVAR, version 2013-11-12). SNV were further filtered as shown in the pipeline (Supplementary material 4). Know variants found in dbsnp129 and dbsnp137 with a MAF > 1% (1000g or ESP6500) were removed. Finally, low frequency SNV and Indel that are suspected to be false positive were systematically inspected with IGV version 2.3.32 (Robinson et al., 2011;Thorvaldsdóttir et al., 2013).

***Drug Sensitivity and Resistance Profiling***

80 oncology compounds of distinct classes, targeting various signaling pathways, including chemotherapeutics, alkylating agents, antimetabolites, antimitotics, antibiotics, kinases inhibitors, immunomodulators, epigenetic targeting agents, proteasome inhibitors, etc) were validated on a panel of leukemia cell lines (unpublished results, and Loosveld et al Oncotarget 2014). The compounds were dissolved in 100% dimethyl sulfoxide (DMSO) and dispensed on tissue culture–treated 96-well plates (Corning). The compounds were plated in four different concentrations in 10-fold dilutions covering a 1,000-fold concentration range, depending on drug activity as evaluated on the panel of leukemia cell lines. The predrugged plates were kept at -20°C (5 µL/well) until needed. The compounds were dissolved with 120 µL of cell culture medium, 25 µL of which were transfered to 75 µL of single-cell suspension (20,000 cells). The plates were incubated in a humidified environment at 37°C and 5% CO2, and after 48 hours, cell viability was measured using CellTiter-Glo luminescent assay (Promega) according to the manufacturer’s instructions with a Centro (Berthold) plate reader. The percentage survival for each data point was calculated and dose–response curves were generated for each of the drugs tested. The dose–response curves were fitted on the basis of a four-parameter logistic fit function defined by the top and bottom asymptote, the slope, and the inflection point (EC 50), and verified manualy. In the curve fitting, the top asymptote of the curve was fixed to 100% viability.

***Mouse xenograft studies***

Briefly, 6 to 10-week-old NOD.Cg-Prkdc ^scid^/J (NSG) mice were obtained from Charles River France or bred in-house and maintained under specific pathogen-free conditions. Animals were injected via tail vein with 10^6^ viable human leukemia cells in 0.1mL sterile PBS. Daily monitoring of mice for symptoms of disease (weight loss >20%, ruffled coat, hunched back, weakness, reduced motility) determined the time of killing for injected animals with signs of distress. Peripheral blood was obtained by retro-orbital bleeding and the presence of ALL blasts was determined by flow cytometry. Human CD19 and CD45 expression was detected by staining with fluorescently conjugated monoclonal antibody (FITC anti-human CD19 antibody, Beckman Coulter and PerCp/Cy5.5 anti-human CD45 antibody, Biolegend). Secondary transplants were conducted by injecting bone marrow cells of primary transplanted mice. Primary ALL cells from second passage mice with equivalent levels of blasts at day 50 were randomized to either vehicle control (citrate buffer, pH 2.75) or experimental groups. NSG mice were treated during 4 weeks by oral gavage with 25mg/kg dasatinib (Sprycel, Bristol-Myers Squibb Eeig; 5 days/ week) or 30mg/kg ponatinib (Iclusig, Ariad Pharmaceuticals; 4 days/ week) where indicated. All experiments were conducted under the supervision of the facility’s Institutional Animal Care and use Committee-approved protocol.

**References to supplementary material**

Adélaïde J, Finetti P, Bekhouche I, Repellini L, Geneix J, Sircoulomb F *et al.* Integrated profiling of basal and luminal breast cancers. *Cancer Res* 2007; **67**: 11565–11575.

Bekhouche I, Finetti P, Adélaïde J, Ferrari A, Tarpin C, Charafe-Jauffret E *et al.* High resolution comparative genomic hybridization of inflammatory breast cancer and identification of candidate genes. *PLoS One* 2011; **6**: e16950.

Cornen S, Guille A, Adelaide J, Addou-Klouche L, Finetti P, Saade MR, *et al.* Candidate luminal B breast cancer genes identified by genome, gene expression and DNA methylation profiling. *PLoS One* 2014; **9**: e81843.

Cortes JE, Kantarjian H, Shah NP, Bixby D, Mauro MJ, Flinn I, *et al.* Ponatinib in refractory Philadelphia chromosome-positive leukemias. *N Engl J Med* 2012; **367**: 2075–2088.

De Braekeleer E, Douet-Guilbert N, Rowe D, Bown N, Morel F, Berthou C, *et al.*. [ABL1 fusion genes in hematological malignancies: a review.](http://www.ncbi.nlm.nih.gov/pubmed/21435002) *Eur J Haematol* 2011; **86**: 361-371.

[De Braekeleer E](http://www.ncbi.nlm.nih.gov/pubmed?term=De%20Braekeleer%20E%5BAuthor%5D&cauthor=true&cauthor_uid=23168614), [Douet-Guilbert N](http://www.ncbi.nlm.nih.gov/pubmed?term=Douet-Guilbert%20N%5BAuthor%5D&cauthor=true&cauthor_uid=23168614), [Guardiola P](http://www.ncbi.nlm.nih.gov/pubmed?term=Guardiola%20P%5BAuthor%5D&cauthor=true&cauthor_uid=23168614), [Rowe D](http://www.ncbi.nlm.nih.gov/pubmed?term=Rowe%20D%5BAuthor%5D&cauthor=true&cauthor_uid=23168614), [Mustjoki S](http://www.ncbi.nlm.nih.gov/pubmed?term=Mustjoki%20S%5BAuthor%5D&cauthor=true&cauthor_uid=23168614), [Zamecnikova A](http://www.ncbi.nlm.nih.gov/pubmed?term=Zamecnikova%20A%5BAuthor%5D&cauthor=true&cauthor_uid=23168614), *et al.*. Acute lymphoblastic leukemia associated with RCSD1-ABL1 novel fusion gene has a distinct gene expression profile from BCR-ABL1 fusion. [*Leukemia.*](http://www.ncbi.nlm.nih.gov/pubmed/23168614) 2013; **27**: 1422-1424.

DePristo MA, Banks E, Poplin R, Garimella KV, Maguire JR, Hartl C, *et al.*. [A framework for variation discovery and genotyping using next-generation DNA sequencing data.](http://www.ncbi.nlm.nih.gov/pubmed/21478889) *Nat Genet* 2011; **43**: 491-498.

Garrison, E. and Marth, G. Haplotype-based variant detection from short-read sequencing. Preprint at [arXiv:1207.3907v2 [q-bio.GN]](http://arxiv.org/abs/1207.3907v2) (2012).

Inokuchi K, Wakita S, Hirakawa T, Tamai H, Yokose N, Yamaguchi H, Dan K. [RCSD1-ABL1-positive B lymphoblastic leukemia is sensitive to dexamethasone and tyrosine kinase inhibitors and rapidly evolves clonally by chromosomal translocations.](http://www.ncbi.nlm.nih.gov.gate2.inist.fr/pubmed/21863287) *Int J Hematol* 2011;**94**: 255-260.

Li H, Durbin R. Fast and accurate short read alignment with Burrows-Wheeler transform. [*Bioinformatics*](http://www.ncbi.nlm.nih.gov/pubmed/19451168) 2009; **25**: 1754-1760.

Mustjoki S, Hernesniemi S, Rauhala A, Kahkonen M, Almqvist A, Lundan T *et al.* A novel dasatinib-sensitive RCSD1-ABL1 fusion transcript in chemotherapy-refractory adult pre-B lymphoblastic leukemia with t(1;9)(q24;q34). *Haematologica* 2009; **94**: 1469–1471.

O’Hare T, Shakespeare WC, Zhu X, Eide CA, Rivera VM, Wang F, et al. AP24534, a pan-BCR-ABL inhibitor for chronic myeloid leukemia, potently inhibits the T315I mutant and overcomes mutation-based resistance. *Cancer Cell* 2009; **16**: 401–412.

Ren M, Qin H, Ren R, Cowell JK. Ponatinib suppresses the development of myeloid and lymphoid malignancies asociated with FGFR1 abnormalities. *Leukemia* 2013; **27**: 32-40.

Robinson JT, Thorvaldsdóttir H, Winckler W, Guttman M, Lander ES, Getz G, Mesirov JP. [Integrative genomics viewer.](http://www.ncbi.nlm.nih.gov/pubmed/21221095) *Nat Biotechnol* 2011; **29**: 24-26.

Sircoulomb F, Bekhouche I, Finetti P, Adélaïde J, Ben Hamida A, Bonansea J *et al.* Genome profiling of ERBB2-amplified breast cancers. *BMC Cancer* 2010; **10**: 539.

Sircoulomb F, Nicolas N, Ferrari A, Finetti P, Bekhouche I, Rousselet E, et al. ZNF703 gene amplification at 8p12 specifies luminal B breast cancer. *EMBO Mol Med* 2011; **3**: 153–166.

Thorvaldsdóttir H, Robinson JT, Mesirov JP. [Integrative Genomics Viewer (IGV): high-performance genomics data visualization and exploration.](http://www.ncbi.nlm.nih.gov/pubmed/22517427)*Brief Bioinform* 2013; **14**: 178-192.

Zhou T, Commodore L, Huang WS, Wang Y, Thomas M, Keats J, *et al.* Structural mechanism of the pan-BCR–ABL inhibitor ponatinib (AP24534): lessons for overcoming kinase inhibitor resistance. *Chem Biol Drug Des* 2011; **77**: 1–11.
