# Supplementary material for: Drug response profiling can predict response to ponatinib in a patient with t(1;9)(q24;q34)-associated B-cell acute lymphoblastic leukemia
Source: Blood Cancer J. 2015 Mar 13;5(3):e292–. doi: 10.1038/bcj.2015.13 (PMC4382656; doi:10.1038/bcj.2015.13)
Supplement: Supplementary Information 2 [file bcj201513x16.docx]

**Supplementary material 8: Figure legends to supplementary material 1-6**

**Supplementary material 1: Expression of fusion *RCSD1/ABL1* and of the normal *RCSD1* and *ABL1* transcripts.**

At the top, RT-PCR was done using RNAs from (a) patient at diagnosis, (b) patient at relapse, (c) XenoG-diagnosis and (d) XenoG-relapse malignant cells with the t(1;9)(q24;q34), and two other leukemia patients that do not present this rearrangement ((e) 13C03176 and (f) 13C03178) (g) normal genomic DNA of PBL and (h) wather were also used as controls They are indicated at the top of each row. Gene expression of *RCSD1/ABL1* and *ABL1/RCSD1* as well as the normal *RCSD1* and *ABL1* transcripts were observed in (a) patient at diagnosis, (b) patient at relapse, (c) XenoG-diagnosis and (d) XenoG-relapse. Sequencing analysis of generated PCR fragments confirms for each of them that the reciprocal t(1,9) rearrangement is associated with *RCSD1/ABL1* gene fusion leading to a splicing between *RCSD1-exon3* and *ABL1-exon4 as well as ABL1-exon3* and *RCSD1-exon4* as previously described (De Braekeleer et al., 2011; Inokuchi K et al, 2011; De Braekeleer et al., 2013).

**Supplementary material 2: *Ex vivo* DRP of a t(1;9)(q24;q34) *RCSD1*-*ABL1* bearing patient.**

Ficoll-purified blood cells obtained from the patient at relapse (April 2014) were exposed for 48h to varying concentrations of the indicated drug compounds (a-h), and cell viability was assessed using CellTiter-Glo luminescent assay (Promega). The data are presented as percent viability after normalization to negative control (DMSO only).

**Supplementary material 3: Genes studied by exome sequencing**

**Supplementary material 4: Comparison of genomic profiles of leukemic cells from patient and corresponding cognate transplants.** Whole genome aCGH profiles are shown for (a) patient at diagnosis, (b) patient at relapse, (c) XenoG-diagnosis and (d) XenoG-relapse. Genomic profiles of patient at diagnosis (a) and at relapse (b) are different (arrows in b). The 6p23-p24 and 13q14.2-q14.3 regional losses (arrows) are only observed in leukemic cells from the patient at relapse (b) and the XenoG-relapse (d). Genomic profiles of patient at diagnosis and at relapse were similar with their corresponding cognate transplants (a with c and b with d, respectively). These results suggest that the clinical evolution of the disease is associated with two regional losses and that the generated animal model reflected the disease as well as its evolution. Chromosome 6 and 13 aCGH profiles (e and f) focusing on chromosome 6p22.3-p24 [11.6-17.6 Mb] and 13q14.2-q14.3 [48.3-53.4Mb] regions are shown for the leukemic cells from patient at diagnosis (green), patient at relapse (brown), XenoG-diagnosis (blue) and XenoG-relapse (red). While the genomic profiles of the patient at diagnosis and from XenoG-diagnosis do not show copy number aberrations (baseline profile), 6p22.3-p24 and 13q14.2-q14.3 losses are observed in the patient at relapse (brown) and the XenoG-relapse (red). They target several genes including potential candidates as *JARID2* (6p22.3), *miRNA15* and *miRNA16* (both on 13q14.2, arrows). These losses might be associated with the t(6;13)(p23;q14) cytogenetically characterized in leukemic cells from the patient at R1 (**g**),

**Supplementary material 5: Primary sequences and combinations of *RCSD1* and *ABL1* primers used to characterize wild-type and fusion transcripts by RT-PCR**

**Supplementary material 6: SNV filtering pipeline**

Scheme showing the SNV filtering process. SNV were filtered based on the variant allele frequency, the Mean quality mapping of observed alternate alleles (MQM), the quality, the strand bias (Fisher's test), and the coverage on each strand.
